# The cost-effectiveness of treatment in chronic HBV non-cirrhotic hepatitis – finite versus long-life therapy

**DOI:** 10.1186/1471-2334-14-S7-P16

**Published:** 2014-10-15

**Authors:** Cristina Popescu, Gabriel Adrian Popescu, Alina Lobodan, Mihaela Rădulescu, Anca-Ruxandra Negru, Roxana Petre, Violeta Molagic, Cristina Covaliov, Adriana Manea, Victoria Aramă

**Affiliations:** 1 National Institute for Infectious Diseases "Prof. Dr. Matei Balş", Bucharest, Romania; 2 Carol Davila University of Medicine and Pharmacy, Bucharest, Romania

## Background

Although the ideal end point of chronic HBV hepatitis therapy is HBsAg loss, a realistic end point is the induction of sustained virological remission. The definitions of virological responses vary according to therapeutic regimen: viral load <2000 IU/mL after interferon (IFN) regimens and undetectable HBV-DNA during nucleoside/nucleotide analogues (NNA) regimens. Objective: To compare the direct costs of medication between two therapeutic strategies: NNA versus NNA after IFN in non-cirrhotic patients without contraindications for IFN.

## Methods

We made a cost simulation analysis, in order to establish the best therapeutic strategy in HBV hepatitis. The rate of response after IFN therapy was about 40%. We theoretically compared the treatment costs for two groups of 100 patients: group 1 treated with entecavir 0.5 mg/day and group 2, treated one year with IFN and then with entecavir in non-responders to IFN. In Romania the cost of IFN is 220 Euro/dose and the cost of entecavir 0.5 mg is 410 Euro/month.

## Results

IFN cost for one patient who received 48 weeks of therapy is 10,560 Euro. For 100 patients the cost is 1,056,000 Euro. The cost of entecavir for one patient, per year is 4920 Euro. For 100 patients the cost of therapy per year is 492,000 Euro. If the IFN response is 20%, for 100 analyzed patients, 80 patients will be subsequently treated with entecavir. If entecavir will be recommended for 5 years the costs are: in group 1 – 2,460,000 Euro for 100 patients and in group 2 – 2,574,960 Euro (1,000,560 Euro for IFN plus 1,574,400 Euro for 80 patients treated 4 years with entecavir). The costs in these two groups are similar; the use of IFN is not cost-effective. If entecavir will be recommended for 10 years the costs are: in group 1- 4,920,000 Euro for 100 patients, in group 2 – 4,542,960 Euro (1,000,560 Euro for IFN plus 3,542,400 Euro for 80 patients treated 9 years with entecavir). A similar analysis for 40% response to IFN, shown that the supplementary cost in group one is 278,640 Euro for 5 years and 1,262,640 Euro for 10 years.

## Conclusion

We need real life studies in order to appreciate the rate and the durability of immune response after IFN therapy. If this rate is 20%, the use of IFN is cost-effective only if entecavir will be used for 10 years or more. For 40% response the use of IFN seems to be cost effective regardless the duration of entecavir therapy.

